# Serologic and genotypic characterization of hepatitis B virus in HIV-1 infected patients from South West and Littoral Regions of Cameroon

**DOI:** 10.1186/s12985-016-0636-x

**Published:** 2016-10-21

**Authors:** Tshifhiwa Magoro, George Gachara, Lufuno Mavhandu, Emmaculate Lum, Helen K. Kimbi, Roland N. Ndip, Pascal Bessong

**Affiliations:** 1 HIV/AIDS & Global Health Research Program, Department of Microbiology, University of Venda, Private bag X5050, Thohoyandou, 0950 Limpopo South Africa; 2 Department of Medical Laboratory Sciences, Kenyatta University, Nairobi, Kenya; 3 Department of Zoology and Animal Physiology, Faculty of Science, University of Buea, Buea, Cameroon; 4 Department of Biological Sciences, Higher Teachers’ Training College, University of Yaounde, Yaoundé, Cameroon; 5 Department of Medical Laboratory Science, Faculty of Health Sciences, University of Bamenda, Bamenda, Bambili Cameroon; 6 Department of Biochemistry and Microbiology, Faculty of Science and Agriculture, University of Fort Hare, Alice, South Africa; 7 Department of Microbiology and Parasitology, University of Buea, Buea, Cameroon

**Keywords:** Hepatitis B virus, HIV/HBV co-infection, HBV genotypes, Phylogenetic analysis

## Abstract

**Background:**

HBV and HIV share similar transmission routes. Concurrent infection with the two viruses usually results in more severe and progressive liver disease, and a higher incidence of cirrhosis, liver cancer and mortality. Further, this co-infection may lead to cross-resistance between HIV and HBV drugs and increased liver injury, either due to direct hepatotoxicity or drug-related immune-reconstitution hepatitis. These challenges necessitate continuous surveillance for HBV among HIV infected individuals to guide patient management. We conducted this study to understand the serologic and genotypic characteristics of HBV among HIV/HBV infected patients in South West and Littoral Regions of Cameroon.

**Methods:**

Plasma samples were screened for HBsAg, HBeAg, Anti-HBs and anti-HBc using ELISA followed by DNA extraction from all HBsAg positive samples. A 366 bp region covering the overlapping surface/polymerase gene was amplified by a nested PCR and the product sequenced using Big Dye sequencing chemistry. The resulting sequences were then analyzed for genotypes and both escape and drug resistance mutations.

**Results:**

Of the 455 samples in this study, 25.5 % (*n* = 116) were HBsAg positive and 46 of these had their DNA successfully amplified. Genotype E was found in 32 samples (69.6 %) and genotype A in the rest of the samples. Escape mutations associated with failure of diagnosis (Y100C, R122K and Q129H) and with vaccine escape (Q129R and T131N) were detected in varying frequencies in the population. Polymerase mutations implicated in resistance to lamivudine and other ʟ-nucleoside analogues were detected in seven patients (15.2 %), while all the samples lacked mutations associated with resistance to adefovir and tenofovir.

**Conclusions:**

These findings suggest the endemicity of HBV and the predominance of genotypes A and E in the study population. Also, drug resistance findings support the use of tenofovir based ART regimens among HIV/HBV co-infected persons. There is need for continuous HBV screening and monitoring in HIV infected individuals in these regions.

## Background

Hepatitis B virus (HBV) [Family *Hepadnaviridae*, Genus *Orthohepadnavirus*] is estimated to infect more than 300 million people worldwide and is a common cause of liver disease and liver cancer [1]. The virus contains a partly double-stranded DNA genome with approximately 3200 base pairs. It replicates via an RNA intermediate anti-genome sequence, encoding a potentially error-prone polymerase enzyme with both reverse transcriptase and DNA polymerase activities [2]. HBV infection is associated with a wide spectrum of clinical manifestations. The outcome of acute infection may range from asymptomatic hepatitis to fulminant liver failure. Failure of viral clearance following acute infection may result in inactive carriage or chronic hepatitis which can progress to both cirrhosis and hepatocellular carcinoma (HCC) [1]. The toll of approximately 1 million deaths from chronic liver disease and hepatocellular carcinoma per year is a clear demonstration of the global health problem posed by this virus [3].

The diagnosis of HBV infection and its associated disease is based on a constellation of clinical, biochemical, histological, and serologic findings [1]. Routinely, it is diagnosed by the presence of the hepatitis B surface antigen (HBsAg) and antibodies to the hepatitis B core antigen (anti-HBc). HBsAg may be detected as early as 1–2 weeks or as late as 11–12 weeks after exposure. Individuals with HBsAg in their serum have overt HBV infection, but do not necessarily have active liver disease; its persistence is considered a marker of chronicity. Hepatitis B e antigen (HBeAg) is a surrogate marker of HBV replication and correlates with the presence of high HBV DNA levels and infectivity. Anti-HBc usually appears in the acute phase of HBV infection and persists for a long time after virus clearance while the presence of anti-HBs with anti-HBc usually indicates a past, resolved infection. In the absence of HBsAg, serum anti-HBs indicate protective immunity against HBV acquired by vaccination (anti-HBc-negative) or natural infection (anti-HBc-positive) [4].

Due to the lack of proof-reading activity of DNA and RNA dependent DNA polymerase, nucleotide mis-incorporation occurs during HBV replication. This is responsible for the emergence of different HBV genotypes and subtypes [5]. These genotypes are defined by inter-genotypic differences of more than 7.5 % in the complete HBV genome. Currently, HBV isolates are classified into 10 genotypes (A–J) and several subtypes, with a differential geographic distribution [6]. Besides differences in geographical distribution, the clinical significance of the genotypes has also been demonstrated. Several studies have shown that genotypes play an important role in treatment management and also in disease prognosis [7, 8]. Information on genotypes is therefore important in identifying patients who are at an increased risk of adverse outcomes and in choosing optimal therapy.

Both HBV and the human immunodeficiency virus (HIV) share common routes of blood-borne and sexual transmission, but they differ in efficiency of transmission and in their geographic distribution [9]. In patients co-infected with HBV and HIV, it has been demonstrated that the HBV X-protein (HBx) super-induces ongoing HIV-1 replication and HIV-1 long-term repeated transcription by synergizing with *tat*- protein and with T-cell activation signals [10]. These findings indicate that HBx could contribute to a faster progression to AIDS in HBV/HIV-co-infected individuals [11]. In both viruses, lamivudine (3TC), a nucleoside analogue is widely used as part of the treatment [12]. The major limitation in the use of lamivudine is the selection of resistant mutants, which affect the tyrosine– methionine–aspartate (YMDD) motif of the HBV DNA polymerase. It has been shown that when lamivudine-resistant variants emerge, HBV DNA levels increase, liver enzyme levels may rise, and the resulting hepatitis can be fatal in a minority of patients. In addition, the bulk of available data suggest that the benefit in preventing progression of liver disease is substantially diminished with the presence of lamivudine-resistant HBV [13]. Furthermore, the use of lamivudine in an HIV/AIDS treatment regimen, as the only anti-HBV component is an unintended monotherapy against HBV in HIV/HBV infected patients, with the potential to select for HBV lamivudine resistance.

Several studies have demonstrated high rates of Hepatitis B and C and HIV infections among HIV infected patients in Cameroon [14, 15]. However, studies in this region focusing on genotypic characterization, analysis of HBV vaccine escape mutants and lamivudine resistance have only started to emerge [16]. We thus sought to investigate the prevalence and genotypic profile of hepatitis-B infection among HIV-infected individuals in South West and Littoral regions of Cameroon.

## Methods

### Study setting

**Fig. 1:**
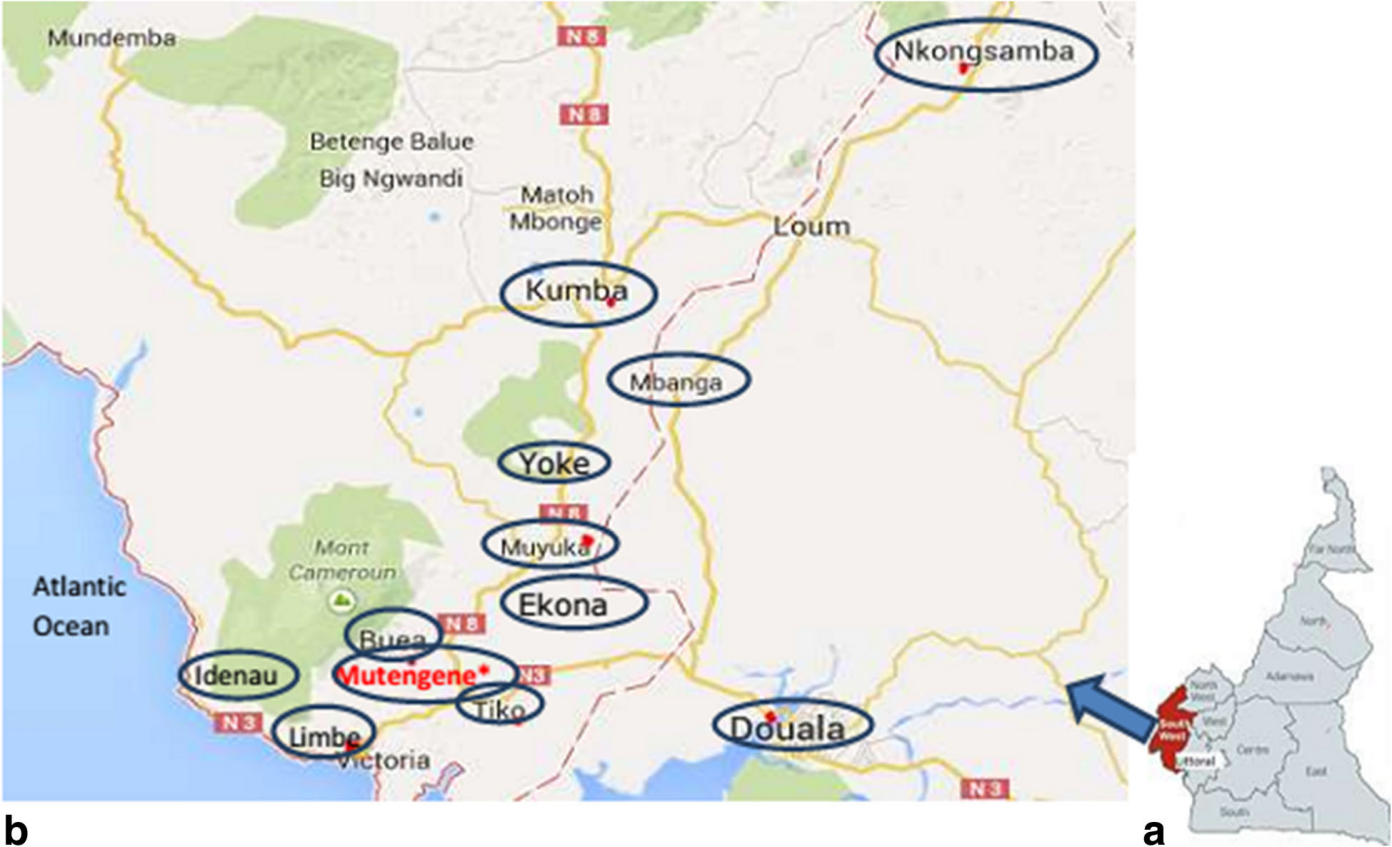
Panel **a**: The map of Cameroon showing the South West region (*highlighted*), from where most of the study participants originated from. Panel **b**: The towns (*circled blue*) from where the study participants originated from. The Mutengene Baptist Health Centre (*indicated in red, with an asterix*) is located in the South West region. (Adapted from Google maps, accessed on 21 June 2015 21:18 pm)

The study participants were outpatient clients of the Mutengene Baptist Health Centre located in the South West Region of Cameroon. The Health Centre offers HIV / AIDS treatment, prevention, medical, spiritual and psychosocial care in the Tiko Health District. The hospital attends to at least 8000 patients per month. Patients come from various towns such as Buea, Limbe, Tiko, Kumba in the South West Region, and Douala, Nkongsamba, in the Littoral Region often because of the good quality of care accorded in this facility. These towns are shown in Fig. 1.

### Study population and ethical considerations

This was a retrospective laboratory-based study and a total of 455 archived HIV positive plasma samples were used. The study protocol was approved by the institutional review board of the Cameroon Baptist Health Board, Cameroon (IRB2012-01) and the Research Ethics Committee of the University of Venda, South Africa (SMNS/14/MBY/21/2110). Written informed consent was obtained from all study participants or their legal guardians prior to their recruitment.

### Sample collection and processing

Five millilitres of venous blood was collected once from each consenting HIV positive adult individual using EDTA vacutainer tubes between February and December 2013. Two ml of blood was collected from young children. Blood was centrifuged for 5 min at 3578Xg. Following centrifugation, plasma was aspirated aseptically and aliquoted into sterile labelled cryotubes and stored at -80°c. These were then transported on dry ice to the HIV/AIDS & Global Health Research laboratories at the University of Venda, South Africa where they were stored at -80°c until used.

### HBV serologic markers

All plasma samples were screened using a commercially available enzyme-linked immunosorbent assay (ELISA) for the presence of HBsAg (Bioelisa, Biokit, Barcelona, Spain). This kit utilizes a sandwich ELISA format using guinea pig anti-HBs antibodies coated to microplate wells which act as the capture antibody and goat anti-HBs antibodies marked with peroxidase which serve as conjugate antibodies. HBsAg positive samples were then screened for HBeAg by ELISA (DRG Instruments GmbH, Germany) while HBsAg negative samples were also screened for anti-HBc and anti-HBs (DRG Instruments GmbH, Germany).

### DNA extraction

DNA from all HBsAg positive samples was extracted from 100 μl of plasma using the Quick-gDNA mini prep (Zymo research, USA) according to the manufacturer’s instructions. The extracted DNA was amplified immediately after extraction or stored at −20 °C for subsequent amplification.

### Polymerase chain reaction for HBV surface/polymerase gene

A nested PCR was performed in order to amplify the overlapping surface/polymerase gene covering nucleotides 403–768 from the EcoR1 site as described previously [17]. The first round reaction was conducted in a 50 μl volume containing 10 mM Tris-HCI pH 8.3, 50 mM potassium chloride, 0.2 mM dNTP mix, 2.5 mM magnesium chloride, 0.2 ng/μl of each primer, and 2 units of *Taq* polymerase (Applied Biosystems, PE, Italia). The samples were subjected to 35 cycles involving denaturation at 95 °C for 1 min, annealing at 55 °C for 1 min, and extension at 72 °C for 1 min. Five μl of the first round PCR product was used as a template for the nested PCR under the same reaction conditions, but performing only 20 cycles. The PCR products were then resolved by 1.5 % agarose gel electrophoresis stained with ethidium bromide. PCR Amplicons were then purified using QiAquick PCR Purification Kit (Qiagen, Hilden; Germany) according to the manufacturer’s instructions.

### Sequencing

Purified PCR products were directly sequenced at Inqaba Biotech (Pretoria, South Africa) according to the Sanger protocol. Contiguous nucleotide sequences (contigs) were assembled from resulting forward and reverse reactions using the SeqMan Pro® module of the Lasergene (version 8.1.5) sequence analysis software suite (DNASTAR. Madison, WI.).

### Sequence analysis

The resulting nucleotide sequences were aligned using the Clustal W program implemented in MEGA 6.06 [18]. They were also translated and checked for HBsAg mutations in the S gene and drug resistance associated mutations in the P gene. A Maximum Likelihood (ML) phylogenetic tree with a bootstrap of 3000 replicates was constructed utilizing the General Time Reversible (GTR) model using MEGA 6.06 [18]. Frequency estimates of evolutionary divergence between nucleotide sequences were then estimated using the Kimura 2-parameter model [19] implemented in MEGA 6.06 [18]. Since the 366 bp fragment sequenced in this study was too short to give resolution for the sub genotypes in the phylogenetic tree (but sufficient to determine the genotypes), the geno2Pheno database [20] was used for their prediction. The sequences from this study have been deposited in GenBank under accession numbers KU900150-KU900195.

### Statistical analysis

The study also determined the impact of the identified HBV genotypes on HIV progression. Mean CD4 cell counts among patients infected by the different genotypes were compared using an independent samples *t* test. This was conducted using SPSS version 20 (IBM, Chicago, IL).

## Results

### Study population

There were a total of 455 HIV positive participants, of whom 343 (75.4 %) were females. The mean age of the patients was 33.8 years (range, 1 to 68 years) while the mean CD4 counts were 406 cells/μl. In terms of their HIV clinical staging, 127 were in clinical stage 1, 166 in stage 2, 198 in stage 3 and 89 in stage 4. A total of 157 participants were not yet on ART at the time of sample collection. Majority of those on ART were on Zidovudine, Lamivudine and Nevirapine.

### Serology

The HBsAg was positive in 116/455 patients resulting in a 25.5 % prevalence. Of the 116 HBsAg positive patients, 90 (26.2 %) were females and 26 (23.2 %) were males while only 9 (7.8 %) were children below 14 years. Samples positive for HBsAg (*n* = 116) were also tested for HBeAg, and 15.7 % (16/102) were positive (14 samples were not screened due to insufficient volumes). Among the HBeAg positive samples, 25 % (4/16) were from children aged below 14 years. Samples that were negative for HBsAg (*n* = 339) were tested for anti-HBs and anti-HBc, of which 13.3 % (44/331) were positive for anti-HBs, and 36.3 % (120/331) were positive for anti-HBc (8 samples were not screened due to insufficient quantity). 20.8 % (69/331) of the patients were positive for both anti-HBc and anti-HBs.

### Prevalent HBV Genotypes

**Fig. 2:**
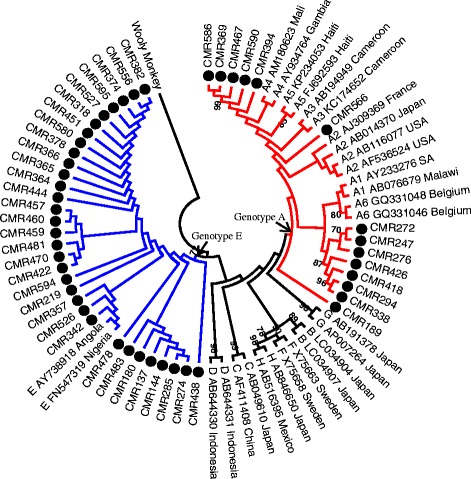
A maximum likelihood rooted phylogenetic tree of HBV sequences from HBV/HIV co-infected individuals from Cameroon. The tree also contains selected global sequences covering the gene region 403–768 from the EcoR1 site. The 46 viruses characterised in this study are indicated with a black circle and fall into genotypes E and A. All reference sequences are labeled with their genotype/sub genotype, accession number and country of origin. Bootstrap values >70 % are shown. The tree is rooted with the Wooley monkey virus sequence

HBV DNA was successfully amplified in 41 % (*n* = 48) of the HBsAg positive samples. Sequencing of the overlapping surface/polymerase gene was successful for 46 out of these 48 HBV-DNA positive samples. Phylogenetic analysis revealed two prevalent genotypes namely A and E. HBV genotype E was identified in 69.6 % (*n* = 32) and HBV genotype A in 30.4 % (*n* = 14) of the participants. Figure 2 shows the phylogenetic relationships of the studied viruses. Of the 14 genotype A viruses, 13 were categorized as sub genotype A1 and 1 as sub genotype A2 by Geno2Pheno analysis. HBV genotype E infected patients had a slightly higher mean CD4 cell count (390 cell/μl) compared to genotype A infected patients (304 cell/μl). However, this difference was not statistically significant (*p* = 0.486).

### HBV genetic diversity

Analyses of nucleotide sequence identity among HBV genotype A from this study indicate that they were 94–100 % similar with an average identity of 96.9 %. Among genotype E, the sequence identity was 97.8 – 100 % with an average identity of 99.4 %.

### HBsAg escape mutants

Mutations within the major hydrophilic region (MHR) of the S gene and which are associated with poor reactivity in serological assays and also immune escape were described. A total of 16 mutations occurring in at least three samples within this region were noted. The most prevalent mutations observed were found either in the ‘a’ region (L127P, S140T and S143T) or the B cell epitope and its surrounding region (G159A, F161Y and A168V). Generally, more mutations were observed within genotype A than genotype E. This is shown in Table 1.

**Table 1:** Mutations found within the major hydrophilic region of the HBV isolated from HBV/HIV co-infected individuals

Mutation	Frequency	Genotype
Y100C	3	A
S114T	13	A
I120L	3	E
R122K	12	A
L127P	14	A
Q129H	3	E
T131K	3	A
T131N	11	A
S140T	14	A
S143T	15	A (14), E (1)
G159A	19	A (12), E (7)
F161Y	14	A
E164G	3	E
E164D	6	A
S167L	4	A
A168V	14	A

### Drug resistant mutations

HBV polymerase mutations associated with resistance to lamivudine and other drugs used in treatment were determined. These mutations were detected in seven patients; six among genotype A and one among genotype E. Mutations rtV173L, rtL180M, rtM204V conferring resistance to lamivudine and other ʟ-nucleoside analogues were identified in six patients while one patient had the rtL180M + rtM204V + rtT184S mutations associated with resistance to both ʟ-nucleoside analogues and entecavir. Three of the patients with lamivudine resistant HBV had no prior exposure to the drug as part of HIV treatment. Mutations conferring resistance to tenofovir and adefovir were not detected in the patients. These results are summarized in Table 2.

**Table 2:** Drug resistant conferring mutations among the study population

Sample ID	Genotype	Drug resistance associated mutations	Lamivudine Experience (months)
272CMR	A	rtV173L, rtL180M, rtM204V	Yes (51)
276CMR	A	rtV173L, rtL180M, rtM204V	Yes (4)
247CMR	A	rtV173L, rtL180M, rtM204V	Naive
438CMR	E	rtL180M, rtT184S, rtM204V	Naive
369CMR	A	rtV173L, rtL180M, rtM204V	Yes (24)
467CMR	A	rtV173L, rtL180M, rtM204V	Yes (48)
586CMR	A	rtV173L, rtL180M, rtM204V	Naive

## Discussion

In the present study, the prevalence of HBsAg among the study participants was found to be 25.5 %. This sero-prevalence is higher than that from recent studies in Cameroon [14, 16, 21]. In these studies, study participants were largely drawn from one town either Yaoundé or Buea. However, it is comparable to the 20.4 % obtained in a study involving pregnant mothers in northern Cameroon [22]. This confirms the hyper endemic nature of HBV in this country. It should also be noted that the high HBsAg sero-prevalence in this study is in coherence with published data within central sub-Saharan Africa. In this region the reported HIV/HBV frequency varies from 0 to >28.4 % [23]. Furthermore, it is important to note that HBV sero-prevalence differs intranationally in some countries with areas of high endemicity.

Children aged 14 years and below had the lowest HBsAg prevalence (7.8 %) in comparison to the other age groups. On the other hand, an HBeAg positivity of 44.4 % (4/9) among children aged below 14 years resulting in a prevalence of 3.9 % (4/102) was documented. Considering that horizontal infection during early childhood is the main route of transmission in high prevalence communities [24], the low HBsAg prevalence in children might be due to the integration in 2005 of the HBV vaccine into the expanded program on immunization (EPI) in Cameroon. It is thus expected that most of the children in the 1–14 years old age group might have been vaccinated. Of concern however is the HBeAg prevalence in this group. Since these children are likely to have been vaccinated and yet had evidence of viral replication, we postulate that either they were more than 9 months old by the time the vaccination was rolled out and were thus not vaccinated or that they were infected with immune escape mutants. Since this early infection may lead to chronic hepatitis and increased risk of liver cancer, this observation warrants further studies.

HBsAg is a marker of active HBV infection. However, HBV DNA was successfully amplified only in 41 % of the HBsAg-positive patients. This is not surprising as inactive HBsAg carriers (diagnosed by absence of HBeAg and undetectable or low levels of HBV DNA) have been shown to form the largest group in chronic HBV infected patients [25]. It has also been shown that HBsAg levels are not always associated with serum HBV DNA levels since HBsAg synthesis can occur independent of HBV replication [26]. Several studies from the same region have reported HBV DNA amplifications of 47 % from HBsAg positive samples [22, 27]. The phylogenetic analysis of the overlapping surface/polymerase gene covering nucleotides 403–768 from the EcoR1 of HBV in this study reveals the predominance of genotype E in the study population. This observation is in agreement with previous studies that have shown the endemicity of this genotype to the ‘genotype E crescent’, which spans from Senegal to Namibia in the South and to the Central African Republic in the East [28, 29]. HBV genotype A was also identified in this study albeit at a lower frequency than genotype E. This trend has also been shown recently [16]. In contrast, several studies have shown the dominance of genotype A in Cameroon [28, 30]. Since both genotype A and E circulate in this country, their relative distribution has been shown to vary depending on the cohort being studied [22].

Concerning the genetic diversity among the identified genotypes, this study observed that genotype E was more conserved with a diversity of 0.6 % compared to 3.1 % in genotype A. Genotype A is significantly heterogeneous and more diverse in Africa suggesting an African origin. This diversity has occasioned its further classification into several sub genotypes and variants [5]. The low intra-genetic diversity of genotype E compared to other genotypes has been described previously [31]. This finding coupled with its presence in West Africa and absence in the Americas in most studies has led to the suggestion that the introduction of genotype E into the human population is a recent event [5]. However, some studies have suggested that its evolutionary history is not as recent as previously thought.

Mutations in the HBsAg are known to allow escape from neutralizing antibodies and also lead to no or poor reactivity with serological assays. Most of these mutations occur in the major hydrophilic region spanning amino acid 99–169 within which there is the ‘a’ determinant (amino acid 120 to 147), the most antigenic part of the S gene [32]. We thus sought to investigate the mutations in this region. In agreement with the genetic diversity results, more mutations were observed among genotype A than E sequences. There were three escape mutations identified which were previously associated with diagnostic failure (Y100C, R122K and Q129H) and two associated with vaccine escape (Q129H and T131N). The reason some mutations previously linked with diagnostic failure were detected in this study might be because the ELISA kit (Bioelisa, Biokit, Barcelona, Spain) used in this study has been shown to detect some of these mutants [33]. We however hope to investigate in the near future mutations in the HBsAg negative samples. The prevalence of vaccine escape mutations was found to be low in this population. Considering that HBV vaccination was incorporated into the Cameroon national EPI in 2005, this is in agreement with the observation that such mutations develop slowly [34].

Different HBV variants or mutants, i.e., viral *quasi species*, are selected within the same host in response to antiviral therapy during the course of infection. This coupled with the fact that primary resistance to any individual drug appears to confer at least some degree of cross-resistance to other drugs presents a significant clinical challenge since remaining treatment options are limited [35]. This study observed previously described drug mutations in seven patients, three of whom had no experience with ARVs. The replacement of methionine in the YMDD catalytic site motif by valine (rtM204V) in the seven patients is associated with resistance to lamivudine and other ʟ-nucleoside analogues. The mutation rtL180M found together with the rtM204V mutation in these patients is associated with resistance to telbivudine and also with enhanced replication [35]. The rtV173L mutation found in six of these patients is known to compensate for replication defects of lamivudine resistant HBV mutants [36]. The rtL180M + rtT184S + rtM204V triple mutation found in one patient suggests resistance to entecavir, lamivudine and other ʟ-nucleoside analogues. Importantly, no resistance mutations associated with the acyclic nucleoside phosphonates namely adefovir and tenofovir were identified. This supports the recommendation by the WHO that tenofovir be made available in the Cameroonian AIDS program for use in HIV/HBV co-infected patients [27].

Three of the seven patients with drug resistant HBV were ARV naïve. This observation in which resistance occurs in ARV naïve patients has also been made previously [37, 38]. It is not clear whether the HBV in these patients actually developed *de novo* lamivudine-resistant mutations or that the patients were infected with lamivudine-resistant viruses that persisted. Some possible explanations for the presence of lamivudine-resistant HBV in the untreated Cameroonian HIV/HBV co-infected patients could be suggested. In a country where lamivudine is the backbone of HIV treatment, we may be constrained to speculate that this may arise from infection with a lamivudine resistant HBV strain acquired from an HIV infected individual currently on treatment but also co-infected with HBV. Overall, these results emphasize the need for sporadic HBV drug resistance screening among HIV/HBV co-infected patients before therapy initiation.

The lack of HBV and HIV viral load data together with liver enzyme levels in this study preclude our ability to correlate the results reported here with clinical outcomes. Furthermore, since this study focused only on HBsAg positive samples, it is possible that more genotypes would have been identified. This is because occult infection (HBV DNA in the absence of HBsAg) is known to occur. Nevertheless, this study provides added insights into the circulating HBV genotypes and variants in this region.

## Conclusions

In conclusion, our results confirm the endemicity of HBV in South West and Littoral regions of Cameroon and the circulation of genotypes E and A. In line with the WHO recommendations, this study supports tenofovir based regimens in the treatment of HBV in HIV/HBV co-infected persons. There is need for continuous monitoring of HBV drug resistance in this population and more studies comparing HBV in HIV infected and uninfected individuals are recommended.
